# Comparability of the small RNA secretome across human biofluids concomitantly collected from healthy adults

**DOI:** 10.1371/journal.pone.0229976

**Published:** 2020-04-10

**Authors:** Scott M. Langevin, Damaris Kuhnell, Jacek Biesiada, Xiang Zhang, Mario Medvedovic, Glenn G. Talaska, Katherine A. Burns, Susan Kasper

**Affiliations:** 1 Division of Epidemiology, Department of Environmental Health, University of Cincinnati College of Medicine, Cincinnati, OH, United States of America; 2 Cincinnati Cancer Center, Cincinnati, OH, United States of America; 3 Division of Biostatistics and Bioinformatics, Department of Environmental Health, University of Cincinnati College of Medicine, Cincinnati, OH, United States of America; 4 Division of Environmental Genetics & Molecular Toxicology, Department of Environmental Health, University of Cincinnati College of Medicine, Cincinnati, OH, United States of America; 5 Division of Environmental & Industrial Hygiene, Department of Environmental Health, University of Cincinnati College of Medicine, Cincinnati, OH, United States of America; Institut de Biologie Moleculaire et Cellulaire, FRANCE

## Abstract

Small extracellular vesicles (sEV) are nano-sized (40–150 nm), membrane-encapsulated vesicles that are released by essentially all cells into the extracellular space and function as intercellular signaling vectors through the horizontal transfer of biologic molecules, including microRNA (miRNA) and other small non-coding RNA (ncRNA), that can alter the phenotype of recipient cells. sEV are present in essentially all extracellular biofluids, including serum, urine and saliva, and offer a new avenue for discovery and development of novel biomarkers of various disease states and exposures. The objective of this study was to systematically interrogate similarities and differences between sEV ncRNA derived from saliva, serum and urine, as well as cell-free small ncRNA (cf-ncRNA) from serum. Saliva, urine and serum were concomitantly collected from 4 healthy donors to mitigate potential bias that can stem from interpersonal and temporal variability. sEV were isolated from each respective biofluid, along with cf-RNA from serum. sEV were isolated from the respective biofluids via differential ultracentrifugation with a 30% sucrose cushion to minimize protein contamination. Small RNA-sequencing was performed on each sample, and cluster analysis was performed based on ncRNA profiles. While some similarities existed in terms of sEV ncRNA cargo across biofluids, there are also notable differences in ncRNA class and ncRNA secretion, with sEV in each biofluid bearing a unique ncRNA profile, including major differences in composition by ncRNA class. We conclude that sEV ncRNA cargo varies according to biofluid, so thus should be carefully selected and interpreted when designing or contrasting translational or epidemiological studies.

## Introduction

There has been surging interest in recent years in studying and exploiting circulating small extracellular vesicles (sEV)–which include exosomes and small microvesicles–as biomarkers for malignant diseases [1–4], non-malignant diseases [5–8], and environmental exposures [9, 10]. sEV are nano-sized (40–200 nm), membrane-encapsulated vesicles that are released by cells as part of normal physiology—as well as malignant or pathologic processes -into the extracellular space [11] where they function as intercellular signaling vectors through the horizontal transfer of biologic molecules, including non-coding RNA (ncRNA).

Small ncRNA are a heterogeneous group of ncRNA less than 200bp length that include microRNA (miRNA), Y RNA [12], transfer RNA (tRNA) [13], small nucleolar RNA (snoRNA) [14], vault RNA (vtRNA) [15], signal recognition particles (SRP RNA) [16]–including 7SL RNA–and small nuclear RNA (snRNA) [17] and 7SK RNA [18]. Some ncRNA classes, such as miRNA, are involved in post-transcriptional regulation of gene expression [19], while others play roles in RNA splicing [20] and other aspects of RNA transcription, processing and translation [18, 21–23], thereby having the capacity to impact cellular phenotype. sEV containing ncRNA are secreted by cells into the extracellular space, where they can fuse with membranes of adjacent cells to regulate normal physiological and pathological states [24] or enter into circulation or other body fluids, such as saliva or urine.

Human serum is the acellular liquid fraction of blood absent of the clotting factors and is comprised of 95% water, with the remaining 5% consisting of suspended or soluble molecules including proteins, lipids, electrolytes, nutrients, and cell-free nucleic acids [25], as well as circulating extracellular vesicles [26]. Human saliva is comprised of 99% water with the remaining 1% being a mixture of other biomaterials [27], including extracellular vesicles [28]. The majority of the molecular content found in whole (unstimulated) saliva originate in the submandibular glands (~65%), with another 20–30% being transported from blood capillaries, and the remainder coming from local cellular and extracellular materials that are shed into the oral lumen. By comparison, urine is comprised of 90–96% water, which is filtered out from the bloodstream via the kidneys and excreted via the urethra [29]. Thus, both saliva and urine contain water, biomolecules and particles, including sEV, which derive from serum and local sources.

Serum, urine and saliva each bear a unique set of advantages and disadvantages in terms of collection for clinical and epidemiologic studies. Saliva is a simple, non-invasive, cost-effective biospecimen that can be easily collected in the clinical setting but can be difficult to obtain from individuals with saliva production issues (i.e. xerostomia)–a common issue, particularly among elderly adults due to systemic disease [30] or radiation therapy to the head and neck [31]. Urine is another easily collected, non-invasive biofluid, albeit a little more challenging than saliva in terms of methods of collection and storage. However, urine yields significantly higher sample volumes, making it easier to obtain higher concentrations of sEV for analysis. In contrast, serum, plasma or whole blood collection involves a minimally-invasive blood draw–although still relatively easy to obtain. Blood offers several advantages due to its (1) common availability through biorepositories and large longitudinal cohort studies, with the latter allowing for prospective validation; and (2) relative ease of collection as a biofluid, including from patients with severe xerostomia. While several studies have assessed differences in cell-free small ncRNA (cf-ncRNA)–a heterogeneous mix of extracellular RNA from a variety of sources–across various biofluids from healthy individuals [32–35], none to date have specifically interrogated differences in sEV ncRNA cargo by biofluid type. Thus, the objective of this study was to comprehensively profile sEV ncRNA cargo across concomitantly collected serum, saliva and urine—as well as serum cf-ncRNA—in order to systematically assess similarities and differences between these biospecimens while mitigating potential bias that can stem from interpersonal and temporal variability.

## Material and methods

### Study participants and biofluid collection

We concomitantly collected saliva, urine and serum from 4 healthy Caucasian non-smoker donors (2 male and 2 female) matched on age (+/- 3 years) and sex, with a median age of 51.5 years (range: 39–65 years). Samples were collected in that order, with serum obtained last to avoid potential confounding from an acute immune response due to the needle stick. Subjects were not asked to fast prior to sample collection. Each subject provided a sample of clean catch urine that was frozen at -80°C in 10 mL aliquots. Saliva (2 mL) was collected from each subject using a Saliva Exosome Collection and Preservation Kit (Norgen Biotek, *Thorold*, *ON*). Blood was collected in 2 × 8 mL Vacutainer^™^ SST^™^ Serum Separation Tubes from each subject using a butterfly needle. Blood was allowed to clot at room temperature for 30 minutes, tubes were centrifuged at 1,500×*g* for 15 minutes to separate the serum, and serum was removed and stored in 1 mL aliquots at -80°C. All subjects gave written informed consent in accordance with the Declaration of Helsinki. The protocol was approved by the University of Cincinnati Institutional Review Board.

### sEV isolation by differential ultracentrifugation

Urine and saliva were isolated via differential ultracentrifugation using the protocol for viscous fluids with a 30% sucrose cushion to remove non-EV-associated lipoproteins or other protein aggregates, as described by Thery et al [36]. Briefly, each saliva (2 mL) and urine (10 mL) sample was centrifuged for 30 minutes at 2,000×*g* at 4°C to remove any cells or cellular debris, after which the resultant supernatant was centrifuged for 45 minutes at 12,000×*g* at 4°C to remove any smaller debris or cellular organelles. The supernatant was then spun in a L8-60M ultracentrifuge (Beckman Coulter, *Brea*, *CA*) using a 70Ti fixed-angle rotor for 75 minutes at 160,000×*g* at 4°C. The supernatant was discarded, pellet was resuspended in 20 mL phosphate-buffered saline (PBS), filtered through a 0.2μm filter, gently pipetted on top of 4 mL Tris/sucrose/D2O solution (30% sucrose cushion), and centrifuged for 75 min at 100,000×g at 4°C using a 70Ti fixed-angle rotor. A 5 mL syringe fitted with an 18-G needle was used to collect approximately 3.0 mL of the sucrose cushion from the side of the ultracentrifuge tube. The aspirate was then transferred to a fresh ultracentrifuge tube, diluted to 60 mL with PBS, and centrifuged for 70 minutes at 100,000×*g* at 4°C using a 45Ti fixed-angle rotor. The sEV pellet was resuspended in 100 μL PBS and stored at -80ºC for downstream analysis. Total RNA was extracted from sEV isolates using the miRNeasy Micro kit (Qiagen, *Valencia*, *CA*) according to the manufacturer’s suggested protocol.

Serum sEV were isolated using a previously described differential ultracentrifugation protocol [37]. Briefly, serum (1 mL) was diluted with equal volume of PBS and centrifuged for 30 minutes at 2,000×*g* at 4°C. The supernatant was transferred to a new tube and centrifuged for 45 minutes at 12,000×*g* at 4°C. The supernatant was removed, PBS was added to bring the total volume to 20 mL, and it was gently pipetted on top of 4 mL Tris/sucrose/D2O solution (30% sucrose cushion) and centrifuged for 75 minutes at 100,000×*g* at 4°C with an Optima L-100K ultracentrifuge using a 70Ti fixed-angle rotor. A 5 mL syringe fitted with an 18-G needle was used to collect approximately 3.5 mL of the sucrose cushion from the side of the ultracentrifuge tube. The aspirate was then transferred to a fresh ultracentrifuge tube, diluted to 60 mL with PBS, and centrifuged for 70 minutes at 100,000×*g* at 4°C using a 45Ti fixed-angle rotor. The sEV pellet was resuspended in 100 μL PBS and stored at -80ºC for downstream analysis. Total RNA was extracted from sEV isolates using the miRNeasy Micro kit (Qiagen, *Valencia*, *CA*) according to the manufacturer’s suggested protocol.

### Isolation of serum cell-free RNA

Samples were lysed and phases separated using Trizol^™^ LS (Thermo Fisher Scientific, *Waltham*, *MA*), which was added to serum aliquots (1 mL) at a 3:1 ratio, centrifuged for 5 minutes at 12,000×*g* at 4°C, and the supernatant was incubated for 5 minutes to allow for dissociation of the nucleoprotein complex. cf-RNA was purified using the Direct-zol^™^ RNA micro prep kit (Zymo Research, *Irvine*, *CA*) according to the manufacturers suggested protocol for biological fluids.

### Nanoparticle tracking analysis and sEV characterization

sEV were quantified and characterized in accordance with the minimal experimental guidelines suggested by the International Society for Extracellular Vesicles (ISEV) [38].The sEV size range and concentration for each isolate was determined via nanoparticle tracking analysis (NTA) using a NanoSight NS300 instrument (Malvern, *Worcestershire*, *UK*). sEV isolates were diluted 1:100 in PBS, with the instrument set to camera level 14 and detection threshold = 5. sEV were visualized/imaged with a JEM-1230 transmission electron microscope (TEM; JEOL, *Tokyo*, *JP*), and the presence of EV-associated tetraspanins (CD9 or CD81) and EV binding protein (TSG101) was also be confirmed by Western blot [39]. Additional methodological details are provided in the Supplementary Material.

### Small RNA-sequencing

Small RNA library preparation and sequencing performed by the University of Cincinnati Genomics, Epigenomics and Sequencing Core. To prepare the library, the NEBNext small RNA sample library preparation kit (New England BioLabs, *Ipswich*, *MA*) was used with ~5 ng of total RNA determined by Bioanalyzer RNA 6000 Pico Kit (Agilent, *Santa Clara*, *CA*) in a 5 μL solution as input, following the manufacturer’s suggested protocol excepting a modification of library size selection to increase small RNA detection sensitivity and specificity. After 15 cycles of PCR for indexing and library enrichment, an equal-volume of 10 μl PCR mix (library without size selection) per sample together with the same volume of the negative control were pooled, followed by DNA cleanup using DNA Clean & Concentrator (Zymo Research, *Irvine*, *CA*) and mixed with 135 and 319 bp custom-made ladders targeting the library 16–200 nt small RNA cDNA insert. Next, precise size selection of the 135–319 bp library via 2.75% agarose gel electrophoresis was performed, library concentration was measured by qPCR using the NEBNext Library Quant Kit (New England BioLabs) on a QuantStudio 5 Real-Time PCR System (Thermo Fisher Scientific). Quantified libraries were clustered onto a flow cell at the concentration of 15 pM using the TruSeq SR Cluster Kit v3 (Illumina), and sequenced for 51 cycles using TruSeq SBS kit on a HiSeq 1000 system (Illumina) to generate a few million reads. Based on the sequencing read number from each sample, an equal-read number pool from the PCR mix was calculated via volume adjustment of the PCR mix. Finally, the same procedure for the second round of sequencing was performed to generate expected number of reads for final data analysis.

### Bioinformatic analysis of small RNA-seq data

Samples were demultiplexed and aligned to the human genome (GRCh38/hg38) using STAR RNA-seq aligner [40]. Small ncRNA (Mt_rRNA, Mt_tRNA, miRNA, misc_RNA, tRNA, rRNA, scRNA, snRNA, snoRNA, sRNA, scaRNA) were quantified based on the M24 release of GENCODE [41, 42] summarizeOverlaps from the GenomicAlignments R package with option NH = 1, which removes all multimapping reads and retains only uniquely aligned reads [43]; ribosomal RNA (rRNA) were filtered out prior to analysis. Post-alignment quality control (RNA-SeQC) was performed to assess number of abundant small RNA and degree of replication between samples [40]. Differential analysis based on counts was performed for all transcripts pre-filtered with rule-of-thumb 3 counts in n samples across all samples, where n is number of samples in smallest groups, using the Bioconductor package edgeR [44, 45]. Statistical significance of differential expression was determined based on FDR-adjusted p-values, where Q < 0.1 [46]. Cluster analysis of differentially expressed small RNA was performed using the Bayesian infinite mixture model [47], and the results were displayed in a heatmap based on reads per kilobase per million mapped reads (RPKM). The small RNA-sequencing data was deposited in the Gene Expression Omnibus (GEO; GSE122621).

## Results

The average particle concentration for the serum, urine and saliva sEV isolates, as estimated via Nanoparticle Tracking Analysis, was 6.95x10^10^ particles/mL, 8.85x10^10^ particles/mL and 8.42x10^10^ particles/mL, respectively. The presence of sEV in the isolates was further confirmed by TEM and Western blot (**S1 Fig** and **S2 Fig**). The median number of reads per sample from small RNA-seq was 40,484,270 (interquartile range (IQR): 34,882,680–43,777,722) with a median read length of 18 bases (IQR: 17.0–18.3). A complete description of aligned reads is provided in **S1 Table**.

**Fig 1:**
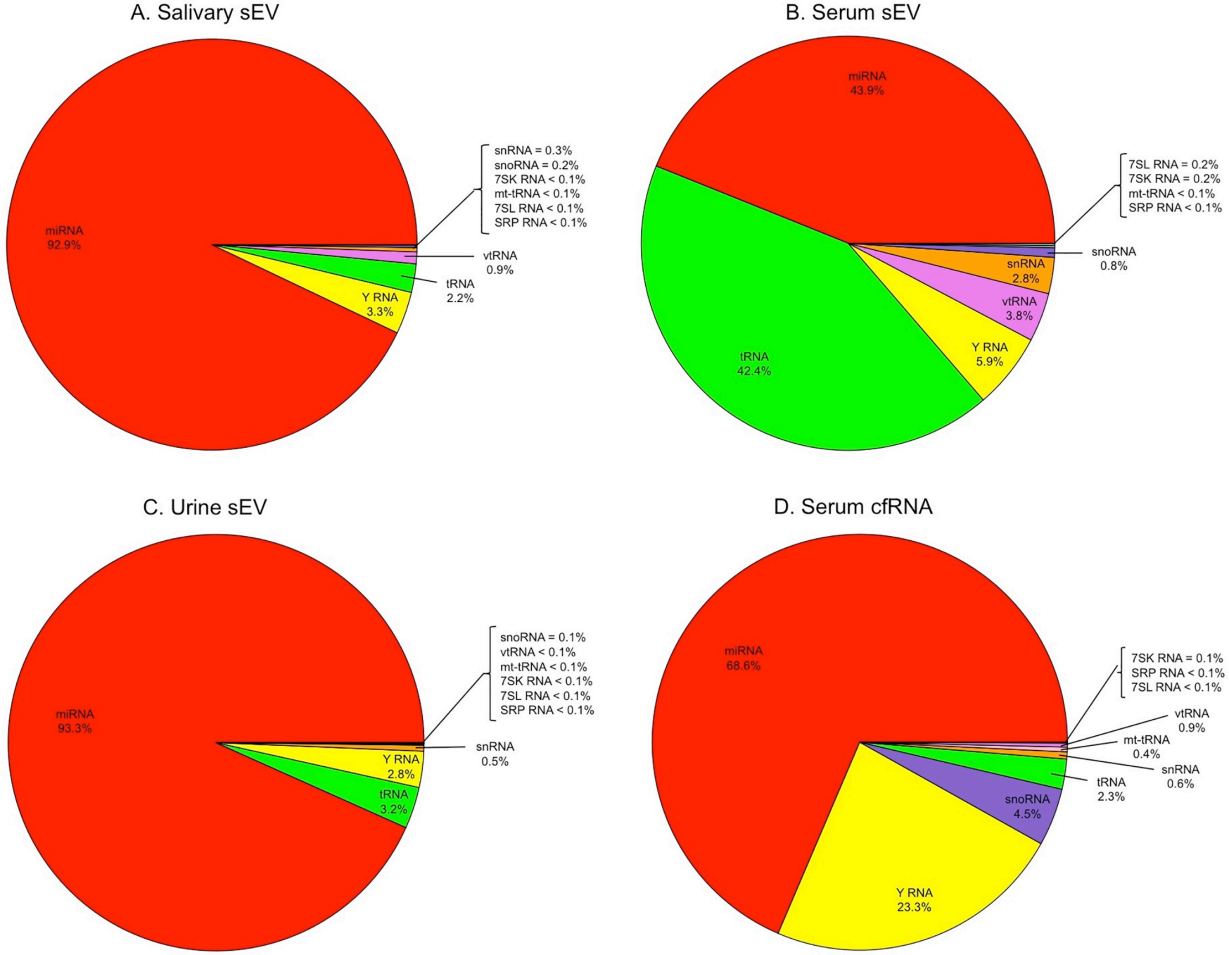
Distribution of uniquely aligned small non-coding RNA species detected in (A) salivary small extracellular vesicles (sEV), (B) serum sEV, (C) urinary sEV, and (D) serum cell-free RNA.

There were 633 unique small ncRNA transcripts with at least 3 reads detected across samples. Although miRNA accounted for the majority of small ncRNA detected in each sample, the relative fractions of ncRNA classes varied substantially according to biofluid source (**Fig 1**). While miRNA comprised 92.9% and 93.3% of uniquely aligned small ncRNA sequences detected in salivary and urinary sEV, respectively, it comprised a smaller, albeit still majority, fraction of serum cf-ncRNA (68.6%), and serum sEV (43.9%). While tRNA only made up 2.2% and 3.2% of salivary and urinary sEV small ncRNA cargo, it accounted for 42.4% of small ncRNA in serum sEV. Interestingly, the high tRNA content was not reflected in serum cf-ncRNA, where it only made up 2.3% of small ncRNA. Conversely, while Y RNA made up 2.8% to 5.9% of sEV small RNA cargo, it comprised 23.3% of small ncRNA in serum cf-ncRNA. There was also a much higher fraction of snoRNA in serum cf-ncRNA (4.5%) compared to the sEV isolates, for which made up from 0.1% − 0.8%. Also worth noting was the relatively higher fractions of snRNA (2.8%) and vtRNA (3.8%) in serum sEV compared to those from saliva and urine.

The ten most highly secreted ncRNA for each respective source accounted for 78% of all saliva sEV ncRNA, 79% for urine sEV, 56% for serum sEV, and 61% for serum cf-ncRNA (**Table 1**). Three small ncRNA transcripts were among the top 10 for all biosample types (*miR-30D*, *miR-148A*, and *RNY1*). Saliva and urine shared the most similarities in terms of top secreted ncRNA, having 7 of 10 top transcripts in common (*miR-148A*, *miR-99A*, *miR-200A*, *miR-200B*, *miR-30D*, *miR-27B*, and *RNY1*). Serum sEV and cf-RNA also shared several common top secreted ncRNA transcripts with 6 of 10 overlapping between the two (*let-7I*, *let-7G*, *miR-30D*, *miR-148A*, *miR-126*, and *RNY1*). A complete table of counts for all 633 ncRNA transcripts detected across samples is provided in **S2 Table**.

**Table 1:** Ten most highly secreted small non-coding RNA detected in each biospecimen type.

	Saliva sEV			Urine sEV			Serum sEV			Serum cfRNA		
Rank	ncRNA Name	ncRNA class	Fraction	ncRNA Name	ncRNA class	Fraction	ncRNA Name	ncRNA class	Fraction	ncRNA Name	ncRNA class	Fraction
1	*MIR148A*	miRNA	37.03%	*MIR10B*	miRNA	27.14%	*30789*	tRNA	14.41%	*RNY1*	Y_RNA	15.83%
2	*MIR375*	miRNA	9.95%	*MIR30A*	miRNA	20.22%	*13038*	tRNA	11.92%	*MIR30D*	miRNA	11.19%
3	*MIR99A*	miRNA	6.28%	*MIR30D*	miRNA	11.55%	*MIRLET7I*	miRNA	6.87%	*MIRLET7I*	miRNA	7.77%
4	*MIR200B*	miRNA	5.56%	*MIR148A*	miRNA	4.34%	*MIR30D*	miRNA	4.41%	*MIR148A*	miRNA	7.67%
5	*MIR30D*	miRNA	4.83%	*MIR99A*	miRNA	3.84%	*MIR148A*	miRNA	4.17%	*MIR320A*	miRNA	5.19%
6	*MIR203A*	miRNA	3.35%	*MIR200B*	miRNA	3.84%	*MIR126*	miRNA	3.69%	*MIR143*	miRNA	3.26%
7	*MIR27B*	miRNA	3.17%	*RNY1*	Y_RNA	2.28%	*MIRLET7G*	miRNA	3.26%	*MIR126*	miRNA	3.16%
8	*RNY1*	Y_RNA	2.71%	*MIR100*	miRNA	2.09%	*VTRNA1-2*	vtRNA	2.89%	*MIRLET7G*	miRNA	2.49%
9	*MIR200C*	miRNA	2.55%	*MIR200A*	miRNA	1.91%	*RNY1*	Y_RNA	2.62%	*MIR191*	miRNA	2.44%
10	*MIR200A*	miRNA	2.43%	*MIR27B*	miRNA	1.76%	*35974*	tRNA	1.95%	*MIR140*	miRNA	2.06%
			77.87%			78.99%			56.19%			61.07%

Total fraction of all detected ncRNA that the top 10 secreted ncRNA account for are displayed at the bottom for each respective biospecimen

Abbreviations

sEV = small extracellular vesicles; miRNA = microRNA; snoRNA = small nucleolar RNA; scaRNA = small Cajal body-specific RNA

**Fig 2:**
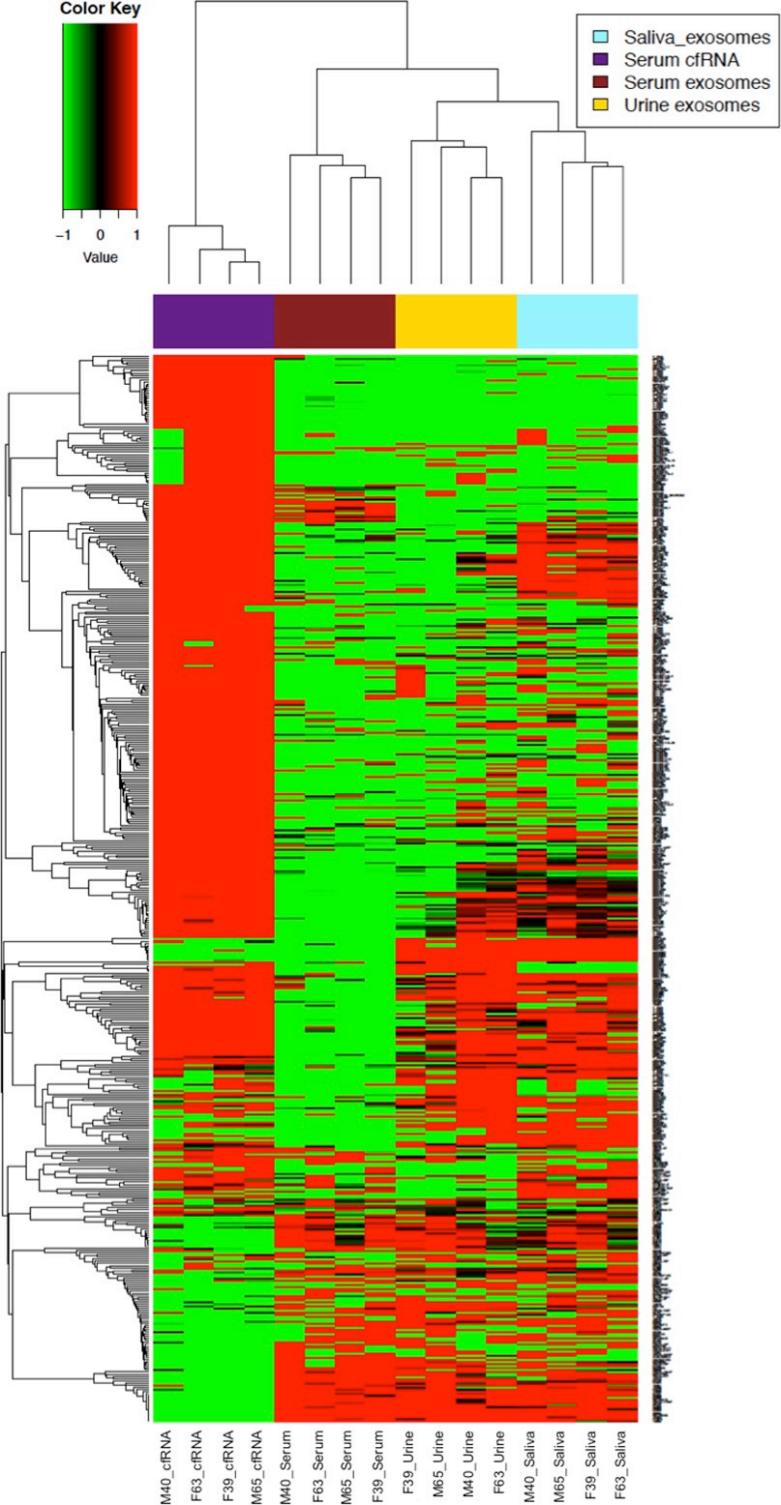
Differential small extracellular vesicle (sEV) small non-coding RNA (ncRNA) cargo across study subjects and biofluids.

There were clear differences in ncRNA profiles according across biospecimens, with samples clustered perfectly according to biosample source (**Fig 2**). The serum cf-ncRNA samples showed the most striking differences in terms of ncRNA profile, with sEV samples being slightly more similar to each other, although clear differences in profiles exist for each sEV source. Urinary and salivary sEV were more similar to one another than they were to serum sEV. No obvious patterns emerged by age or sex.

## Discussion

This study demonstrates notable differences across biofluids concomitantly collected from healthy subjects, notable differences exist, with sEV in each biofluid bearing a unique ncRNA profile. Although saliva and urine were most similar, there are still substantial differences in their respective profiles. Also of major importance in terms of biomarker development is the substantial difference observed between serum sEV and cf-RNA. A question that frequently arises is: “Why not utilize the more easily isolated cfRNA rather go through the additional laborious effort of isolating sEV”? The results of this study underscore that cfRNA and sEV are markedly different with respect to small ncRNA profile and thus should be treated as discrete biomarker sources.

Our study has several major strengths, most notably including concomitant intra-person sample collection, inclusion of a 30% sucrose cushions in the ultracentrifuge isolation protocols to minimize protein contamination [36], and use of small RNA-sequencing for comprehensive characterization of the small ncRNA secretome in saliva sEV, urine sEV, serum sEV and serum cfRNA in healthy subjects. Although the modest sample size could be considered a limitation of this study–particularly compared to studies of sEV cargo across biofluids contained in large repositories–we believe that this is offset by our ability to make within-subject comparisons using concomitantly collected samples, thereby mitigating the issue of inter- and intrapersonal bias stemming from different collection times and conditions. It is also plausible that some technical variability may have been introduced, since sEV RNA (miRNeasy) was isolated using different methodology from cf-RNA (DirectZol). However, the observed differences between cf-ncRNA and sEV-ncRNA are not subtle and are far beyond expected technical variation between small RNA isolation protocols.

Despite modest similarities suggesting some overlap of sEV ncRNA cargo across biofluids, as well as between sEV and cfRNA, the differences were far from insignificant, with each biospecimen bearing its own distinct ncRNA profile. These differences imply that serum, saliva and urine serve as reservoirs of sEV that are generated by separate groups of tissues or cell types in the body. Therefore, determining which type of biofluid to collect may depend on the disease or pathological process being investigated. In summary, the take-home message from this work is that the choice of biofluid for interrogation of sEV ncRNA cargo is critical, and should be selected, calibrated, validated and interpreted according to sample source with great care when designing biomarker studies.

## Supporting information

S1 Fig Representative transmission electron microscopy images (120,000×) of small extracellular vesicle isolates from (A) saliva, (B) serum and (C) urine.
(PDF)

S2 Fig Western blot gel images for exosome-associated tetraspanins CD81 (saliva and urine) and CD9 (serum) and cytosolic protein TSG101 (saliva, serum and urine). (PDF)

S1 Methods (PDF)

S1 Table (XLSX)

S2 Table (XLSX)
