# Supplementary material for: Comparability of the small RNA secretome across human biofluids concomitantly collected from healthy adults
Source: PLoS One. 2020 Apr 10;15(4):e0229976. doi: 10.1371/journal.pone.0229976 (PMC7147728; doi:10.1371/journal.pone.0229976)
Supplement: S1 Fig — Representative transmission electron microscopy images (120,000×) of small extracellular vesicle isolates from (A) saliva, (B) serum and (C) urine. (PDF) [file pone.0229976.s001.pdf]

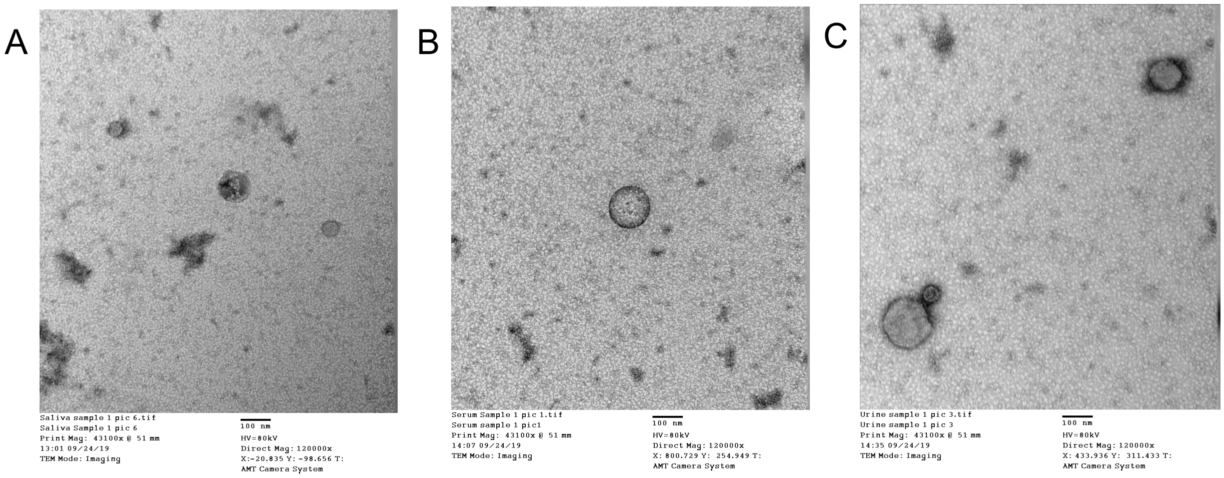

Supplemental Figure S1. Representative transmission electron microscopy images (120,000×) of small extracellular vesicle isolates from (A) saliva, (B) serum and (C) urine.
