# Supplementary material for: Comparability of the small RNA secretome across human biofluids concomitantly collected from healthy adults
Source: PLoS One. 2020 Apr 10;15(4):e0229976. doi: 10.1371/journal.pone.0229976 (PMC7147728; doi:10.1371/journal.pone.0229976)
Supplement: S2 Fig — (PDF) [file pone.0229976.s002.pdf]

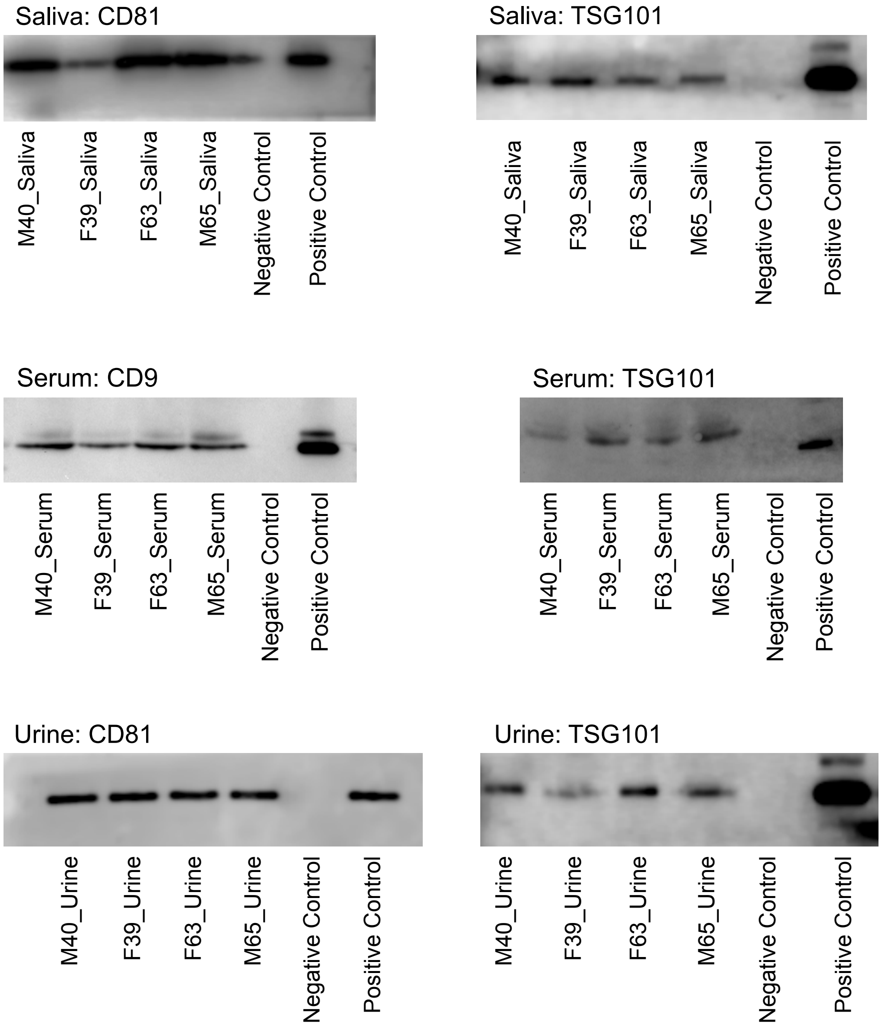

Supplemental Figure S2. Western blot gel images for exosome-associated tetraspanins CD81 (saliva and urine) and CD9 (serum) and cytosolic protein TSG101 (saliva, serum and urine).
