# Supplementary material for: Comparability of the small RNA secretome across human biofluids concomitantly collected from healthy adults
Source: PLoS One. 2020 Apr 10;15(4):e0229976. doi: 10.1371/journal.pone.0229976 (PMC7147728; doi:10.1371/journal.pone.0229976)
Supplement: S1 Methods — (PDF) [file pone.0229976.s003.pdf]

SUPPLEMENTAL METHODS

Western Blot
The cytosolic exosome-associated protein TSG101 was measured for each isolate. The exosome-associated tetraspanin CD81 was measured for saliva and urine; CD9 was measured in serum. Saliva and urine sEV isolates were suspended in PBS; serum sEV isolates were suspended in RIPA Buffer with a proteinase inhibitor cocktail tablet.

CD81
Samples were mixed with 4x Laemmli SDS sample buffer (non-reducing) and run on a NuPAGE 4-12% Bis-Tris gel (Invitrogen) in a mini gel tank (Thermo Fisher Scientific) with MOPS SDS running buffer for 50 minutes at 200V constant. Proteins were transferred onto a PVDF-membrane with Step 1-Transfer buffer on a Pierce Power station (Thermo Scientific) for 7 minutes at 1.3A constant. Membranes were blocked for one hour with 5% milk in TBST before adding CD81 antibody (Abcam; ab79559) at 1:1000 in 5% milk in TBST overnight at 4°C. The membrane was washed 3 times for 5 minutes each in TBST before adding secondary antibody Goat anti-Mouse igG H&L (HRP) (Abcam; ab205719) at 1:3000 in 5% milk in TBST for three hours. Membranes were again washed 3 times at 5 minutes each in TBST. Detection was performed with Super Signal West Femto substrate (Thermo Scientific) on C-DiGit Blot Scanner (Li-Cor) for 12 minutes.

CD9
Samples were mixed with 4x Laemmli SDS sample buffer (non-reducing) and NuPAGE reducing agent (10x), heated to 95°C for 7 minutes and run on a NuPAGE 4-12% Bis-Tris gel (Invitrogen) in a mini gel tank (Thermo Fisher Scientific) with MOPS SDS running buffer with added NuPAGE antioxidant for 50 minutes at 200V constant. Proteins were transferred onto a PVDF-membrane with Step 1- Transfer buffer on a Pierce Power station (Thermo Scientific) for 10 minutes at 1.3A constant. Membranes were blocked for one hour with 5% milk in TBST before adding CD9 antibody (System Biosciences; EXOAB-CD9A-1) at 1:1000 in 5% milk in TBST overnight at 4°C. Membrane were washed 3 times for 5 minutes each in TBST before adding secondary antibody Goat anti-Rabbit HRP (System Biosciences; EXOAB-CD9A-1 included) at 1:10000 in 5% milk in TBST for one hour. Membranes were again washed 3 times for 5 minutes each in TBST. Detection was performed with Super Signal West Femto substrate (Thermo Scientific) on C-DiGit Blot Scanner (Li-Cor) for 12 minutes.

TSG101
Samples were mixed with 4x Laemmli SDS sample buffer (non-reducing) and NuPAGE reducing agent (10x), heated to 95°C for 7 minutes, and run on a NuPAGE 4-12% Bis-Tris Gel (Invitrogen) in a mini gel tank (Thermo Fisher Scientific) with MOPS SDS running buffer and NuPAGE antioxidant gel for 50 minutes at 200V constant. Proteins were transferred onto a PVDF-membrane with Step 1-Transfer buffer on a Pierce Power station (Thermo Scientific) for 10 minutes at 1.3A constant. Membranes were blocked for one hour with 5% BSA in TBST before adding TSG101 antibody (Abcam; ab30871) at 1:1000 in 5% BSA in TBST overnight at 4°C. Membranes were then washed 3 times for 5 minutes each in TBST before adding secondary antibody Goat anti-Rabbit igG H&L (HRP) (Abcam; ab205718) at 1:2000 in 5% BSA in TBST for two hours. Membranes were again washed 3 times at 5 minutes each in TBST. Detection was

performed with Super Signal West Femto substrate (Thermo Scientific) on C-DiGit Blot Scanner (Li-Cor) for 12 minutes.

Transmission Electron Microscopy

sEV were visually confirmed by transmission electron microscopy (TEM) imaging with a JEOL JEM-1230 instrument. To prepare the sample, a drop of 0.1% bovine serum albumin (BSA) was placed on a formvar carbon coated grid for 1 min and then wicked away with a piece of filter paper. We then placed 10 μL of the sEV isolate on the grid for 5 minutes, after which we gently wicked the sample away and added 10 drops of 2% aqueous uranyl acetate (UA) to the grid. The UA was then wicked away and the grid allowed to dry prior to imaging.
